# Acupuncture Points Stimulation for Meniere's Disease/Syndrome: A Promising Therapeutic Approach

**DOI:** 10.1155/2016/6404197

**Published:** 2016-07-28

**Authors:** Jiaojun He, Liyuan Jiang, Tianqiang Peng, Meixia Xia, Huade Chen

**Affiliations:** The Third Clinical Medical College of Zhejiang Chinese Medical University, Hangzhou, Zhejiang 310053, China

## Abstract

*Objective.* This study aims to explore evidence for acupuncture points stimulation (APS) in treatment of Meniere's disease (MD).* Method.* A literature search was conducted in seven databases including EMBASE, Medline, Cochrane Library, Web of Science, CBM, CNKI, and WangFang database and the data analysis was performed by using the RevMan version 5.3.* Results.* 12 RCTs with 993 participants were acquired after the search. The quality of most eligible studies was very low which limited the value of the meta-analysis. Compared with western medicine comprehensive treatment (WMCT), the APS alone or in combination with WMCT had a significant positive effect in controlling vertigo; however, the result was negative in hearing improvement and DHI. No adverse events were reported in the studies.* Conclusion*. The APS might be a promising therapeutic approach for MD. However, the currently available evidence is insufficient to make a definitive conclusion for the poor quality of included studies. More high-quality researches with larger sample size are urgently needed to assess the effectiveness and safety.

## 1. Introduction

Meniere's disease (MD), named after the French physician Prosper Meniere who firstly reported it in 1861 [1], is an idiopathic inner ear disorder characterized by episodic vertigo, fluctuating sensorineural hearing loss, tinnitus, and aural pressure. Some other complaints from patients including drop attack known as otolithic crisis of Tumarkin [2] and nausea [3, 4] always cooccur with the cardinal symptoms. The prevalence in reports ranged from 3.5 to 513 per 100,000 [5] with a slight female preponderance: about 1.89 : 1 in an American investigation [5, 6] and familial clustering, genetic heterogeneity [7, 8]. It is more common in people who are older and white [9] but rare in children [10].

Meniere's disease is a relentless illness [11], which means there would never be an ending through the whole life. The primary disability, vertigo, always accompanied by vomiting, makes the sufferers unable to keep normal posture [12]. Another predominant impact on the quality of life is impaired hearing. The hearing loss appeared in low-frequency at the earlier stage when it comes even without any prevision and goes and then gradually progressed to high-frequency until it developed to profound sensorineural hearing loss or single-sided deafness permanently [13, 14]. What the MD brings is not only physical dysfunction but also the mental problems consisting of anxiety and depression [15, 16]. It seems that there is a vicious cycle between them. The manifestations might be an origin of the unhealthy mental reaction and then the psychiatric comorbidity might well contribute to its pathology [17, 18].

Tons of endeavors have been devoted to the treatment ever since it was reported, but therapeutic progress was so frustratingly slow [19], which should be blamed on the complicated and exclusive mechanism. Until now, there has been no gold standard for treatment that can be adopted as the guideline and the strategies are needed to be individually tailored. The treatment, usually, starts with life-style change, and then there are the etiologic treatments including diuretics, betahistine, intratympanic gentamicin, intratympanic steroids, and surgery [20]. All available therapies, indeed, helped substantial patients. However, not all the sufferers were sensitive to the medications which might produce tolerance or side-effects after a long-term intake [21] or eligible to the surgery. Therefore, complementary and alternative therapy noticed by growing otolaryngology patients [22] might be a good choice for some people.

Acupuncture, a well-known complementary and alternative therapy, has been widely used in China. The symptoms of MD have been observed by Chinese antiquity and have been recorded in Huangdi Neijing [23]; however, the history that acupuncture, moxibustion, and massage were used in otorhinolaryngology could even date back to 5th century BC, much earlier than the time the masterpiece was written [24]. Nowadays, different acupuncture points stimulations (APS) are widely adopted in controlling the vertigo caused by various reasons including MD [25, 26] which made us wonder whether or not APS has some benefits to the sufferers. An analysis was carried out to explore evidence for the utilization of APS in MD.

## 2. Methods

### 2.1. Search Strategy

A strict research protocol was drafted before the work. According to the strategy, databases involving PubMed, EMBASE, Cochrane Library, Web of Science, Chinese BioMedical Literature Database (CBM), Chinese National Knowledge Infrastructure (CNKI), and WangFang data were searched. The studies were published before May 2015, regardless of the striation of language. The key words or free text words and the searching strategies were as follows: (“Meniere's disease” OR “Meniere's syndrome”) AND (“acupuncture” OR “electroacupuncture” OR “acupoint” OR “meridian” OR “auricular therapy” OR “acupressure” OR “acupoint injection” OR “complementary medicine” OR “alternative medicine”) AND (“clinical trial” OR “randomized controlled trial”).

### 2.2. Criteria for Inclusion and Exclusion

Inclusion criteria were as follows: types of studies: randomized controlled trials; types of intervention and control: the main intervention for the experimental group is acupoints stimulations (including mammal acupuncture, scalp acupuncture, ear acupuncture, and auricular-plaster with vaccaria seed, moxibustion, acupoint injection, and acupressure which can be used alone or together) in combination with western medications comprehensive treatment (WMCT). The control group received western medications such as betahistine and other vasodilator, nutritional supports. Types of outcome assessments were the total effective rate assessed by the similar criteria and Dizziness Handicap Inventory (DHI).

Exclusion criteria included the following: (1) duplicated studies and animal experiments; (2) comparison between different acupuncture techniques or acupoints selection; (3) acupuncture in the junction with Chinese herbal medicine.

### 2.3. Data Extraction

According to the inclusion and exclusion criteria, two investigators (Jiaojun He and Liyuan Jiang) independently screened the titles and abstracts and then downloaded the full text if they were potentially eligible for the analysis. The collection of information included the author(s), publish year, diagnostic criteria, sample size, disease course, the acupuncture intervention, control intervention, treatment course, main acupoints, effective criteria, and outcome measurement.

### 2.4. Quality of the Studies

The quality of the included trials was evaluated by two authors independently (Jiaojun He and Liyuan Jiang) in accordance with the risk of bias provided by Cochrane Handle Book 5 which consists of the following 7 items: random sequence generation, allocation concealment, blinding of participants and personnel, blinding of outcome assessment, incomplete outcome data, selective reporting, and other bias. All risks were evaluated as low, high, or unclear. Discrepancies reached an agreement after the discussion with the third reviewer (Huade Chen).

### 2.5. Data Synthesis and Analysis

Meta-analysis was performed by RevMan 5.3 of the Cochrane Collaboration. The outcome was presented as relative ratios (RRs) with 95% confidence intervals (CI) or mean difference with 95% CI. Before the data synthesis and analysis, heterogeneity test was done with the chi-squared test and the Higgins *I* ^2^ test [27]. Random effect models should be used if *I* ^2^ > 50%; otherwise, a fixed effect model should be used. Begg's test and Egger's test were conducted to evaluate publication bias via a funnel-plot when the number of eligible studies was equal to or greater than 10.

## 3. Results

### 3.1. Literature Search

**Figure 1:**
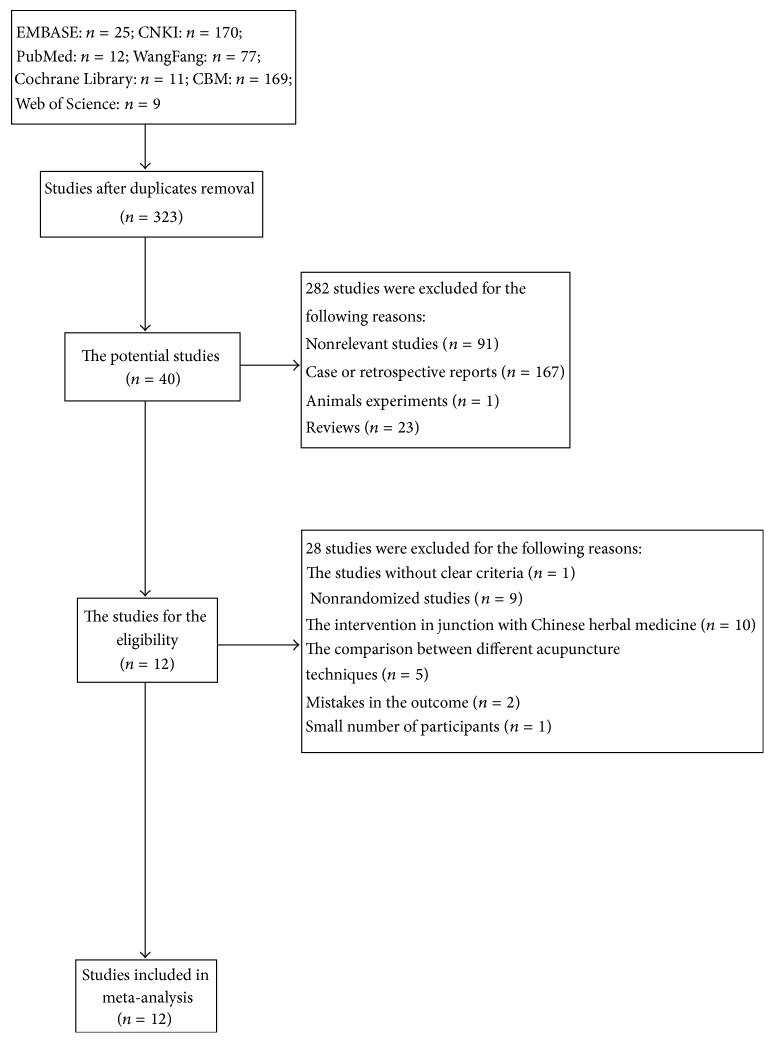
Flowchart of the studies selection process.

The detailed process of the search work was shown in the flowchart (Figure 1). A total of 473 articles we got form the initial search, and 323 of them were left after removing duplicates. And then 282 articles were excluded because they were nonrelevant (*n* = 91), case reports (*n* = 167), animals experiment (*n* = 1), and reviews (*n* = 23). 40 reports with control group remained. One of them was excluded because of lack of the diagnostic criteria, 9 of them were excluded because they were not RCT, 5 of them were excluded for the comparison between different acupuncture techniques, 10 of them were excluded for the junction with Chinese herbal medicine, and 3 of them were excluded for unavailable data and the small number of participants (less than 20). Finally, we included 12 studies for the meta-analysis.

### 3.2. The Basic Characteristics of Included Studies

The basic characteristics and main outcome of the 12 trials were summarized in Tables 1 and 2. All trials [28–40], in which the age range for participants was from 18 to 75 and the disease duration was several days to more than two decades, were conducted in China. The 12 RCTs with clear diagnostic criteria included 993 patients who had typical MD symptoms: 504 participants in the experimental group and 489 patients in the control group.

The interventions included traditional acupuncture, manual acupuncture (MA) in 3 studies [29, 33, 34, 36], MA coupled with moxibustion in two studies [28, 39], techniques in modern acupuncturology containing auricular-stimulation in two reports [30, 37], scalp acupuncture in one study [32], acupoint injection in two trials [31, 38], acupressure in one report [40], or the combination between traditional and modern acupuncture in a study [35]. The main acupoints selected were Baihui (DU20), the top in the studies, Tinggong (SI19), and Fengchi (GB20). The mean treatment time was approximately 10 to 15 days once a day. Two studies [29, 36] mentioned Deqi, an indispensable element for MA, a sort of acid bilge feeling in patients and a sense in doctors which was vividly described as holding a float bobbing up and down when a fish was biting hook.

The follow-up time was 2 months in one report [29], 6 months in another two [28, 38], and 2 years in four articles [32–34, 36], and the rest even did not mention the follow-up. Clinical effective rates were the main outcome in 10 trials [28–38] and the other two [39, 40] employed the DHI.

### 3.3. Risk of Bias Assessment

**Figure 2:**
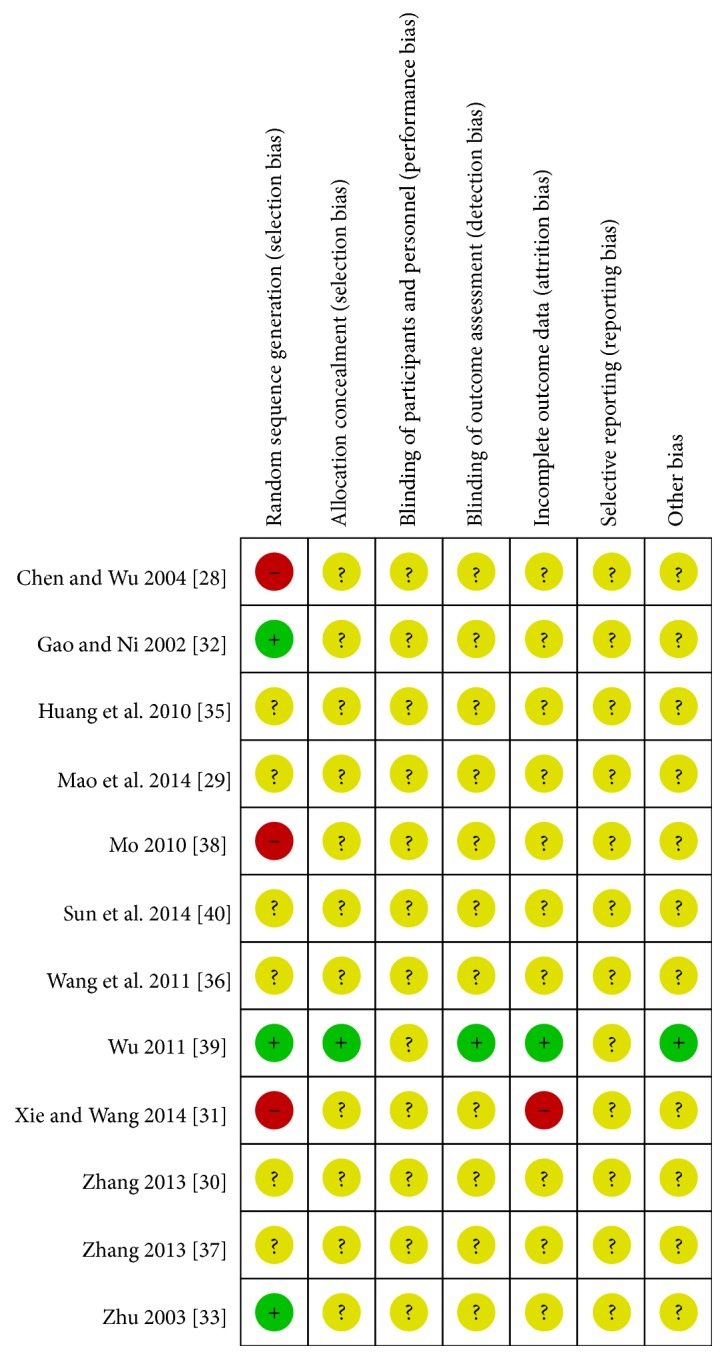
The risk of bias assessment for each included study.

The risk of bias of the included RCTs was summarized in Figure 2.

All studies mentioned randomization; however, the bias, actually, in only 3 studies [32–34, 39] was considered low because of the right random sequence generation from random number table; two of them [28, 31, 38] were high for the visiting sequence, and the information in rest was not enough to make a judgement. One trial [39] used sealed envelope for allocation concealment and proper blinding to outcome, assessed by third party. There were some data missing in a trial [31], but the author did not give relevant reason; therefore the bias was considered high. No reports mentioned that the research was approved by ethics committee and was registered.

### 3.4. Effect Estimates

#### 3.4.1. Total Effective Rate Assessed by TCM Effective Criteria 1994

Four trials adopted effective rate as the outcome by categorization of main symptoms improvement in four levels ((1) clinical cure, (2) markedly effective, (3) effective, and (4) inefficacious), a generally accepted rule in TCM which was performed in 1994. The total effective rate, the sum of the first three items, was the target of the analysis.

**Figure 3:**
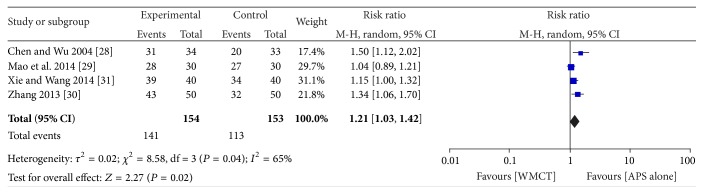
The forest plot of APS alone on total effectiveness assessed by TCM effective criteria 1994.

**Figure 4:**
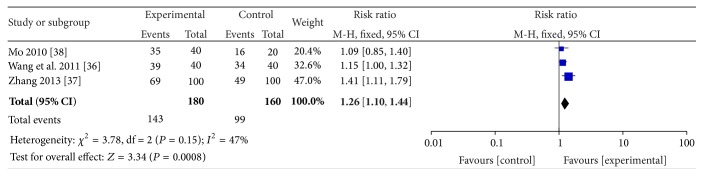
The forest plot of APS plus WMCT on total effectiveness assessed by TCM effective criteria 1994.

Four studies [28–31] compared APS alone with the western medicine comprehensive treatment (WMCT). With significant heterogeneity (*I* ^2^ = 65%, *P* = 0.04), the result yields favours in the APS (RR = 0.21; 95% CI, 1.03–1.42; *Z* = 2.27; *P* = 0.02). Three trials [36–38] showed that APS plus WMCT was significantly better than WMCT (*I* ^2^ = 47%, *P* = 0.15, RR = 1.26; 95% CI, 1.10–1.44; *Z* = 3.34; *P* = 0.0008) (see Figures 3 and 4).

#### 3.4.2. Total Effective Rate Assessed by Chinese Medical Association of Otorhinolaryngology Criteria 1997

**Figure 5:**
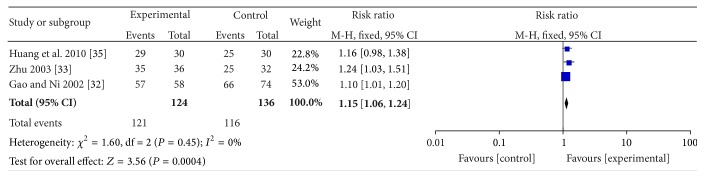
The forest plot of APS plus WMCT on reducing vertigo frequency.

**Figure 6:**
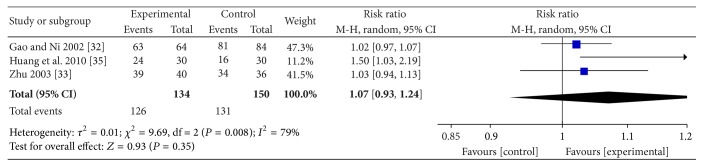
The forest plot of APS plus WMCT on hearing improvement.

3 RCTs [32–35] adopted efficacy standard made by Chinese Medical Association of Otorhinolaryngology, which contained the assessment of vertigo frequency and hearing. In a consequence, the meta-analysis was performed, respectively. As for the vertigo, the result of heterogeneity test showed that *I* ^2^ = 0% and *P* = 0.45 > 0.05, meaning that a fixed effects model should be used. The synthesis results indicated that the APS combined with WMCT had a better effect than WMCT alone (RR = 1.15; 95% CI, 1.06–1.24; *Z* = 3.56; *P* = 0.0004) (Figure 5). As for the hearing function, with significant heterogeneity (*I* ^2^ = 79%; *P* = 0.008), meaning that a random model needed to be adopted, the data did not show significant difference between APS plus WMCT and WMCT alone in the improvement of hearing (RR = 1.07; 95% CI, 0.93, 1.24; *Z* = 0.93; *P* = 0.35) (Figure 6).

#### 3.4.3. DHI after the Interventions

**Figure 7:**
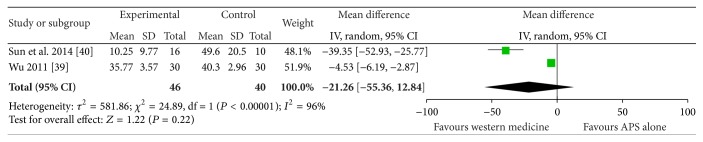
The forest plot of APS alone on DHI.

The score from the questionnaire named DHI was the outcome in the remaining 2 trials [39, 40]. Compared with the WMCT group, the result failed to show a favour in APS group (MD = −21.26; 95% CI, −55.36, 12.84; *P* = 0.22) (Figure 7).

### 3.5. Publication Bias

The number of included studies in each part was less than 10, which was not enough to perform Begg's test, Egger's test, and funnel-plot.

### 3.6. Adverse Events

All the included studies did not describe adverse events during the progress of the treatment, a difficulty in evaluation of the safety of the APS.

## 4. Discussion

To our knowledge, this is not the first time to find evidence for acupuncture used in the remedies of MD. The first one with the conclusion that acupuncture has potential benefits for the person with MD was published in 2011 [41]. Because of the language barrier, the authors just searched one Chinese database which was not very popular in China. After a more comprehensive search work, we made a meta-analysis, but we did not have much progress this time. In our analysis, the APS alone or plus WMCT displayed a positive effect in controlling vertigo but negative in hearing loss and DHI. However, the certain conclusion that APS is effective or is not effective for MD still cannot be settled down due to the poor quality of the included trials.

The quality of methodology in the included trials was very poor. Firstly, the vast majority of the studies failed to describe the details of the production of randomization and allocation concealment. Secondly, the lack of blinding among the patients and caregivers was a common problem in all the studies, which might lead to pronounced bias [42]. Finally, almost all the eligible studies were published in Chinese; if not, the experiment was also conducted in China. Moreover, the positive results highly exist in Chinese reports [43] which led to the publication bias. All the drawbacks might limit the value of the meta-analysis results.

Currently, no special medical remedy can solve the problem of hearing loss very well. The APS was also ineffective in our result. According to our own observation and clinical experience, APS, indeed, had good effect in controlling the vertigo but it was not good in the hearing improvement. The negative result did not mean that APS was completely helpless in the treatment of MD. The negative result, meaning that APS was ineffective in hearing improvement, suggested that the hearing did not change much or even got worse. As long as it was not the worsening one, keeping the existing hearing or delaying the development of hearing loss was not a so bad result for patients.

Tinnitus, an easily negligible symptom, is also a terrible symptom which impacts the patients' quality of life [44]. It did not draw any attentions in our included studies. However, the application of acupuncture in the tinnitus has been in debate for over 40 years [45]. Several systematic reviews [46, 47] could not reach a definitive conclusion owing to the methodological flaws and risk bias. The similar phenomenon happened in our analysis again. Its major responsibility was the lack of proper blinding and sham acupuncture. What made the blinding and sham acupuncture hard to be put into practice was the acupuncture feature that was, naturally speaking, a sort of benign and minimally invasive therapy needed to be manipulated by a specialized doctor. In other words, blinding the performers to the intervention would be hardly possible in clinical trials. And then the blinding and sham acupuncture seemed to be not feasible to the patients who have already experienced acupuncture particularly in China where the population who did not know acupuncture is small.

Supposing the blinding and sham acupuncture has been worked out, the assessment of APS for MD is still a hard nut to crack. Acupuncture as well as the other acupoints stimulation is a patient-centered therapy. The prescription is determined by the syndrome, the degree, and the physical conditions of patients. Consequently, the APS could not display the full capacity in the case of uniform treatment, a conflict with the strict methodology. The only solution to both is collecting the patients with the same disease and physical condition, but it sounds like a story in the Arabian nights.

The sample sizes in eligible trials were relatively small which is likely to overestimate the acupuncture efficacy. Moreover, the number of the included studies was limited and the results can be easily dominated by a single trial, which was a risk to the stability of our result. However, MD should be considered as a rare disease. Although the research focusing on the epidemiology was in blank in China, it was 50 per 100 000 in reports from Japan [48], an Asian nation too, which was much lower than cardiovascular disorders. As a consequence, it would be a very tough work to enroll adequate participants who are eligible to the RCT. Moreover, MD is a mysterious problem and hard to be diagnosed [49], always confused with the vestibular migraine because of the symptom overlap [50], which is also an unfavourable factor to the number of participants.

The measures of stimulating the points in our included studies were quite wide-range which involved near to all the techniques in traditional and modern acupuncture. Based on the same TCM theory, it has to be admitted that there are still some distinctions among them. The different techniques along with the different treatment duration may be responsible for slight or significant heterogeneity that existed in the analysis.

The interventions, combined with two or more techniques, were too complicated to analyze the exact effectiveness of each one. It was, obviously, an undeniable flaw in our meta-analysis. Looking at it, however, from another perspective, it might be a light for the treatment, which might be a daring idea from us or just might be nonsense. MD, currently, without any cure, needs a long-period treatment, which might produce tolerance even without exception to acupuncture. Therefore, the combinations, like the union medicine in hypertension, might strengthen the effects and delay the appearance of tolerance.

MD is a chronic and episodic disease with a remission between two attacks that means that the terrible symptoms can disappear themselves without any medical care. So the follow-up time plays a significant part in the effective assessment. However, the time in most included trials, less than 2 years, was too short to clarify where the effects came from, the effectiveness of APS or self-recovery. Moreover, most studies take the relief of self-reported symptoms as the effective standard rather than the AAO-HNS guidelines [51]. The results collected from self-reported symptoms can be easily affected by subjective emotion and judgement from both sides.

Considering the poor quality of present trials, more future rigorous randomized clinical trials are needed. Researchers should adopt right method of random sequence generation, allocation concealment, and blinding. The data statistics should be reasonable and the number of the dropouts, withdrawals, and the relevant explanations should be described clearly as well as the properly diagnostic and effective criteria and detail about the treatment progress.

## 5. Conclusions

In summary, the analysis results revealed a positive effect in controlling the vertigo but a negative effect in the hearing improvement and DHI. However, the currently available evidence is insufficient to make the conclusion that APS is effective or useless in the therapy of MD for the small scale of the included trials and for the poor quality. More rigorously designed trials are urgently needed to evaluate the validity of APS in the treatment of MD. This is not the first systematic review and also would never be the last one. What we desire is raising attentions to this nonpharmaceutical management, figuring out the shortcomings in present clinical trials, and providing some help to further trials.

## Figures and Tables

**Table 1:** The basic characteristics of included studies.

Study	Country	Study design	Sample size	Age	Disease duration	EC approval
Chen and Wu 2004 [28]	China	RCT	T: 34 C: 33	T: 28–65 C: 28–65	T: 5 days–10 years C: 5 days–10 years	Not reported

Mao et al. 2014 [29]	China	RCT	T: 30 C: 30	T: 25–49 C: 26–49	Not reported	Not reported

Zhang 2013 [30]	China	RCT	T: 50 C: 50	T: 25–63 C: 26–63	T: 3 days–2 years C: 3 days–2 years	Not reported

Xie and Wang 2014 [31]	China	RCT	T: 40 C: 40	T: 25–57 C: 26–63	T: 3 days–10 months C: 2 days–11 months	Not reported

Gao and Ni 2002 [32]	China	RCT	T: 58 C: 74	T: 16–76 C: 16–78	T: 3 days–7 years C: 2 days–7 years	Not reported

Zhu 2003 [33]	China	RCT	T: 40 C: 40	T: 18–76 C: 18–77	T: 2 days–9 years C: 3 days–9 years	Not reported

Huang et al. 2010 [35]	China	RCT	T: 30 C: 30	T: 20–63 C: 20–63	T: 3 months–3 years C: 3 months–3 years	Not reported

Wang et al. 2011 [36]	China	RCT	T: 40 C: 40	T: 20–60 C: 20–60	Not reported	Not reported

Zhang 2013 [37]	China	RCT	T: 100 C: 100	T: 45–76 C: 40–71	T: 3 days–11 years C: 3 days–11 years	Not reported

Mo 2010 [38]	China	RCT	T: 100 C: 100	T: 20–64 C: 20–64	Not reported	Not reported

Wu 2011 [39]	China	RCT	T: 30 C: 30	T: 28–65 C: 28–65	T: 2 years–20 years C: 2 days–20 years	Not reported

Sun et al. 2014 [40]	China	RCT	T: 16 C: 10	T: 20–70 C: 20–70	Not reported	Not reported

*Note.* RCT: randomized controlled trial; T: treatment group; C: control group; EC: ethical committee.

**Table 2:** Interventions and outcome assessment of included studies.

Study	Diagnostic criteria	T (main acupoints)	Control treatment	Treatment duration	Main outcome	Follow-up
Chen and Wu 2004 [28]	TCM effective criteria 1994	MA (DU20, GB8, SI19, GB2, SJ5, GB41, ST36) + moxibustion (DU20)	WMCT (niacin, VB6, ATP injection)	20 days Once a day 20 min	Effective rate	6 months

Mao et al. 2014 [29]	TCM effective criteria 1994	MA: sufficiency syndrome (DU20, GB20, LR3, PC6, SL19); deficiency syndrome (DU20, GB20, BL18, BL23); Deqi	WMCT (oral betahistine)	7 days Once a day 20 min	Effective rate	2 months

Zhang 2013 [30]	TCM effective criteria 1994	Ear acupuncture (kidney, spleen, ear shen men, internal ear)	WMCT (glucose, VB6 solution injection; chlorpromazine tablets, oral oryzanolum)	30 days Keeping for 7 days	Effective rate	Not reported

Xie and Wang 2014 [31]	TCM effective criteria 1994	Acupoint injection (PC6, LR3)	WMCT (niacin, oral VB6)	5 days Once a day	Effective rate	Not reported

Gao and Ni 2002 [32]	Criteria 1997	Scalp acupuncture (MS 6, MS 7) + WMCT	WMCT (buflomedil hydrochloride, hydrochloric acid, Danshen injection)	30 days Once a day Manual operation for 5 min and then a pause for 3 min, 3 times totally	Effective rate	2 years

Zhu 2003 [33]	Criteria 1997	MA (DU20, GB20, SI19) + WMCT	WMCT (glucose, ATP, Danshen injection)	30 days Once a day 30 min	Effective rate	2 years

Huang et al. 2010 [35]	Criteria 1997	MA (DU20, PC20, SI19, ST 36, SI19, SJ21) + moxibustion (DU20) + acupoint injection (GB34) + WMCT	WMCT (gastrodin injection, oral flunarizine)	10 days Once a day 20 min	Effective rate	2 years

Wang et al. 2011 [36]	TCM effective criteria 1994	MA (DU20, GB20, DU16, SJ17, SI19) + WMCT	WMCT (betahistine, Danshen injection), Deqi	30 days Once a day 30 min	Effective rate	2 years

Zhang 2013 [37]	TCM effective criteria 1994	Auricular-plaster (kidney, spleen, ear shen men, internal ear) + WMCT with vaccaria seed	WMCT (oral flunarizine)	12 days Once every two days	Effective rate	Not reported

Mo 2010 [38]	TCM effective criteria 1994	Acupoint injection (ST 40, ST36)	WMCT (anisodamine solution injection, chlorpromazine tablet, oral flunarizine)	Not reported Once a day	Effective rate	6 months

Wu 2011 [39]	DHI	MA (DU20, GB20, LR3, GB12, SJ4, GB2)	WMCT (oral sibelium)	6 days Once a day 30 min	DHI	Not reported

Sun et al. 2014 [40]	DHI	Acupressure (Diaoshi Jifa)	WMCT (Ginkgo injection)	1 day Once a day	DHI	No follow-up

*Note.* MA: manual acupuncture; T: treatment group; C: control group; WMCT: western medicine comprehensive treatment; Criteria 1997: Chinese Medical Association of Otorhinolaryngology criteria 1997.

## References

[1] Meniere P. (1861). Maladie de l’oreille interne offrant les symptôms de la congestion cérébrale apoplectiforme. *Gazette Médicale de Paris*.

[2] Perez-Fernandez N., Montes-Jovellar L., Cervera-Paz J., Domenech-Vadillo E. (2010). Auditory and vestibular assessment of patients with Ménière's disease who suffer Tumarkin attacks. *Audiology and Neurotology*.

[3] Hägnebo C., Andersson G., Melin L. (1998). Correlates of vertigo attacks in Meniere's disease. *Psychotherapy and Psychosomatics*.

[4] Longridge N. S. (1983). The value of nausea and vomiting due to Ménière's disease—a theory. *Journal of Otolaryngology*.

[5] Alexander T. H., Harris J. P. (2010). Current epidemiology of Meniere's syndrome. *Otolaryngologic Clinics of North America*.

[6] Shojaku H., Watanabe Y., Fujisaka M. (2005). Epidemiologic characteristics of definite Ménière's disease in Japan. A long-term survey of Toyama and Niigata prefectures. *ORL: Journal for Oto-Rhino-Laryngology and Its Related Specialties*.

[7] Requena T., Espinosa-Sanchez J. M., Cabrera S. (2014). Familial clustering and genetic heterogeneity in Meniere's disease. *Clinical Genetics*.

[8] Morrison A. W., Bailey M. E. S., Morrison G. A. J. (2009). Familial Ménière's disease: clinical and genetic aspects. *The Journal of Laryngology & Otology*.

[9] Tyrrell J. S., Whinney D. J. D., Ukoumunne O. C., Fleming L. E., Osborne N. J. (2014). Prevalence, associated factors, and comorbid conditions for ménière's disease. *Ear and Hearing*.

[10] Choung Y. H., Park K., Kim C. H., Kim H. J., Kim K. (2006). Rare cases of Ménière's disease in children. *Journal of Laryngology and Otology*.

[11] Semaan M. T., Megerian C. A. (2011). Ménière's disease: a challenging and relentless disorder. *Otolaryngologic Clinics of North America*.

[12] Fujimoto C., Egami N., Kinoshita M., Sugasawa K., Yamasoba T., Iwasaki S. (2013). Factors affecting postural instability in Meniere's disease. *Otolaryngology—Head and Neck Surgery*.

[13] Samy R. N., Houston L., Scott M. (2015). Cochlear implantation in patients with Meniere's disease. *Cochlear Implants International*.

[14] Hansen M. R., Gantz B. J., Dunn C. (2013). Outcomes after cochlear implantation for patients with single-sided deafness, including those with recalcitrant méniére's Disease. *Otology and Neurotology*.

[15] Kanzaki J., Goto F. (2015). Psychiatric disorders in patients with dizziness and Ménière's disease. *Acta Oto-Laryngologica*.

[16] Furukawa M., Kitahara T., Horii A. (2013). Psychological condition in patients with intractable Meniere's disease. *Acta Oto-Laryngologica*.

[17] Sakagami M., Kitahara T., Okayasu T. (2016). Negative prognostic factors for psychological conditions in patients with audiovestibular diseases. *Auris Nasus Larynx*.

[18] Horner K. C., Cazals Y. (2005). Stress hormones in Ménière's disease and acoustic neuroma. *Brain Research Bulletin*.

[19] Foster C. A. (2015). Optimal management of Ménière's disease. *Journal of Therapeutics and Clinical Risk Management*.

[20] Sharon J. D., Trevino C., Schubert M. C., Carey J. P. (2015). Treatment of Menière's disease. *Current Treatment Options in Neurology*.

[21] Băjenaru O., Roceanu A. M., Albu S. (2014). Effects and tolerability of betahistine in patients with vestibular vertigo: results from the romanian contingent of the OSVaLD study. *International Journal of General Medicine*.

[22] Shakeel M., Trinidade A., Ah-See K. W. (2010). Complementary and alternative medicine use by otolaryngology patients: a paradigm for practitioners in all surgical specialties. *European Archives of Oto-Rhino-Laryngology*.

[23] Huppert D., Brandt T. (2016). Descriptions of vestibular migraine and Menière's disease in Greek and Chinese antiquity. *Cephalalgia*.

[24] Yap L., Pothula V. B., Warner J., Akhtar S., Yates E. (2009). The root and development of otorhinolaryngology in traditional Chinese medicine. *European Archives of Oto-Rhino-Laryngology*.

[25] Huang N., Li C. (2012). Recurrent sudden sensorineural hearing loss in a 58-year-old woman with severe dizziness: a case report. *Acupuncture in Medicine*.

[26] Chiu C. W., Lee T. C., Hsu P. C. (2015). Efficacy and safety of acupuncture for dizziness and vertigo in emergency department: a pilot cohort study. *BMC Complementary and Alternative Medicine*.

[27] Higgins J. P. T., Thompson S. G. (2002). Quantifying heterogeneity in a meta-analysis. *Statistics in Medicine*.

[28] Chen J. Y., Wu H. Y. (2004). 34 Cases of Meniere’s disease treated with acupuncture and moxibustion. *Modern Journal of Integrated Traditional Chinese and Western Medicine*.

[29] Mao L. Y., Lu H. J., Shen X. H. (2014). Observation on therapeutic effect of acupuncture on Meniere's disease. *Shanghai Journal of Acupuncture and Moxibustion*.

[30] Zhang Y. H. (2013). 50 Cases of Meniere's disease treated by auricular implantation. *Journal of Gansu College of Traditional Chinese Medicine*.

[31] Xie L., Wang Y. (2014). 60 cases of Meniere's disease treatment by point injection with anisodamine. *Journal of Jiang Xi University of TCM*.

[32] Gao X. P., Ni H. H. (2002). Observation on therapeutic effect of scalp acupuncture on Meniere's disease. *Chinese Acupuncture & Moxibustion*.

[33] Zhu H. X. (2003). 36 cases of Meniere's disease patients treated with acupuncture. *Clinical Journal of An Hui Traditional Chinese Medicine*.

[34] Huang Q. (2009). Fifty cases of vertebrobasilar ischemic vertigo treated by acupuncture. *Journal of Traditional Chinese Medicine*.

[35] Huang C. J., Zhou J. X., Yin W. Y. (2010). Observation on therapeutic effect of acupuncture combined with western medicine on Meniere's disease. *Chinese Journal of Modern Drug Application*.

[36] Wang X., Xia D. K., Cui Y. L. (2011). The clinical observation about the Meniere's disease patients treated by acupuncture combined with western medicine. *Journal of Clinical Acupuncture and Moxibustion*.

[37] Zhang W. (2013). Observation on effect of auricular points embedding in the combination with flunarizine on Meniere's disease. *Chinese Community Doctor*.

[38] Mo L. B. (2010). Observation on effect of acupoints injection with tian ma injection on Meniere's disease. *Journal of Chang Chun University of Traditional Chinese Medicine*.

[39] Wu X. *Clinical research on traditional acupuncture manipulation combined with the moxibustion on BaiHui of Meniere's disease [M.S. thesis]*.

[40] Sun Y.-X., Wang Y., Ji X. (2014). A randomized trial of Chinese Diaoshi Jifa on treatment of dizziness in Meniere's disease. *Evidence-Based Complementary and Alternative Medicine*.

[41] Long A. F., Xing M., Morgan K., Brettle A. (2011). Exploring the evidence base for acupuncture in the treatment of Ménière's syndrome—a systematic review. *Evidence-Based Complementary and Alternative Medicine*.

[42] Hróbjartsson A., Emanuelsson F., Thomsen A. S. S., Hilden J., Brorson S. (2014). Bias due to lack of patient blinding in clinical trials. A systematic review of trials randomizing patients to blind and nonblind sub-studies. *International Journal of Epidemiology*.

[43] Vickers A., Goyal N., Harland R., Rees R. (1998). Do certain countries produce only positive results? A systematic review of controlled trials. *Controlled Clinical Trials*.

[44] Stephens D., Pyykkö I., Yoshida T. (2012). The consequences of tinnitus in long-standing Ménière's disease. *Auris Nasus Larynx*.

[45] Tassinari M., Mandrioli D., Gaggioli N., Roberti Di Sarsina P. (2015). Ménière's disease treatment: a patient-centered systematic review. *Audiology and Neurotology*.

[46] Kim J. I., Choi J. Y., Lee D. H. (2012). Acupuncture for the treatment of tinnitus: a systematic review of randomized clinical trials. *BMC Complementary and Alternative Medicine*.

[47] Liu F., Han X., Li Y., Yu S. (2016). Acupuncture in the treatment of tinnitus: a systematic review and meta-analysis. *European Archives of Oto-Rhino-Laryngology*.

[48] Shojaku H., Watanabe Y., Fujisaka M. (2005). Epidemiologic characteristics of definite Ménière's disease in Japan: a long-term survey of Toyama and Niigata prefectures. *ORL: Journal for Oto-Rhino-Laryngology and Its Related Specialties*.

[49] Vassiliou A., Vlastarakos P., Maragoudakis P., Candiloros D., Nikolopoulos T. (2011). Meniere's disease: still a mystery disease with difficult differential diagnosis. *Annals of Indian Academy of Neurology*.

[50] Lopez-Escamez J. A., Dlugaiczyk J., Jacobs J. (2014). Accompanying symptoms overlap during attacks in Menière's disease and vestibular migraine. *Frontiers in Neurology*.

[51] American Academy of Otolaryngology—Head and Neck Foundation (1995). Committee on Hearing and Equilibrium guidelines for the diagnosis and evaluation of therapy in Meniere's disease. *Otolaryngology—Head and Neck Surgery*.

